# Sex-specific variation in signaling pathways and gene expression patterns in human leukocytes in response to endotoxin and exercise

**DOI:** 10.1186/s12974-016-0758-5

**Published:** 2016-11-10

**Authors:** Asghar Abbasi, Rodolfo de Paula Vieira, Felix Bischof, Michael Walter, Masoud Movassaghi, Nicole C. Berchtold, Andreas M. Niess, Carl W. Cotman, Hinnak Northoff

**Affiliations:** 1 Institute for Memory Impairments and Neurological Disorders (MIND Institute), University of California-Irvine (UCI), Irvine, CA USA; 2 Laboratory of Pulmonary and Exercise Immunology (LABPEI), Nove de Julho University (UNINOVE), Sao Paulo, Brazil; 3 Hertie Institute for Clinical Brain Research and Center of Neurology, University Hospital Tuebingen, Tuebingen, Germany; 4 Agilent Technologies Sales and Services, GmbH & Co. KG, Hewlett-Packard-Strasse 8, 76337, Waldbronn, Germany; 5 Department of Pathology and Laboratory Medicine, University of California—Los Angeles (UCLA), Los Angeles, CA USA; 6 Department of Sports Medicine, University Hospital Tuebingen, Tuebingen, Germany; 7 Zentrum für Klinische Transfusionsmedizin (ZKT) and Institute of Clinical and Experimental Transfusion Medicine (IKET), University Hospital Tuebingen, Tuebingen, Germany; 8 Institute for Memory Impairments and Neurological Disorders (MIND Institute), Gillespie Neuroscience Research Facility, 1113, University of California, Irvine, 92697.4540 USA

**Keywords:** Exercise, Sex differences, Inflammatory genes, LPS stimulation, Toll-like receptors

## Abstract

**Background:**

While exercise effects on the immune system have received increasing attention in recent years, it remains unclear to what extent gender and fluctuations in sex hormones during menstrual cycle influence immunological responses to exercise.

**Methods:**

We investigated mRNA changes induced through exhaustive exercise (half-marathon; pre-exercise and post-exercise [30 min, 3 h, 24 h] on whole blood cultures ± lipopolysaccharide [LPS] [1 h]) with a specific focus on sex differences (men vs women in luteal phase) as an extension of our previous study.

**Results:**

Inflammation related signaling pathways, TLRs, cytosolic DNA sensing and RIG-I like receptors were differentially activated between sexes in LPS-stimulated cultures. Genes differentially regulated between sexes included TNIP-1, TNIP-3, IL-6, HIVEP1, CXCL3, CCR3, IL-8, and CD69, revealing a bias towards less anti-inflammatory gene regulation in women compared to men. In addition, several genes relevant to brain function (KMO, DDIT4, VEGFA, IGF1R, IGF2R, and FGD4) showed differential activation between sexes. Some of these genes (e.g., KMO in women, DDIT4 in both sexes) potentially constitute neuroprotective mechanisms.

**Conclusions:**

These data reveal that the exercise-induced change in gene expression might be gender and menstrual cycle phase dependent.

**Electronic supplementary material:**

The online version of this article (doi:10.1186/s12974-016-0758-5) contains supplementary material, which is available to authorized users.

## Background

While the impact of exercise on immune system functions has in general received increasing attention in recent years, it remains unclear to what extent gender and fluctuations in sex hormones during menstrual cycle influence immunological responses to exercise.

There is accumulating evidence that sex differences have profound effects on the immune system. Overall, the activated NK cell and cytotoxic T cell response is higher in female as compared to male athletes. In addition, women in the second phase of their menstrual cycle show less anti-inflammatory response to exhaustive exercise as compared to men [1]. The differing immune responses to exercise seem to be largely related to fundamental differences in the immune systems of males and females. In general, while men are more prone to bacterial, viral and parasitic infection and sepsis, women have a higher prevalence of several autoimmune diseases, including rheumatoid arthritis (RA) and multiple sclerosis. These fundamental differences between the immune systems of males and females suggest that gonadal hormones and genes of the X chromosome (approximately 1000 genes) may contribute to this sex difference [2].

Little is known about the sex- and menstrual phase- specific differences of the immune response, particularly in the context of endurance exercise. However, understanding sex-specific adaptations to exercise and how immune responses in females may vary across their hormonal cycling, particularly in the context of pathogen contact, are important for optimizing athletic performance and maintenance of health. Previously, we investigated sex- and menstrual phase-dependent differences in immunological responses to exercise (1 h treadmill at 93% of individual anaerobic threshold) in a microarray study of peripheral blood mononuclear cells (PBMCs) [3]. Gene expression of 789 genes was assessed immediately after exercise and compared between women in the luteal phase of their menstrual cycle, the same women in the follicular phase of their menstrual cycle, and men. This study revealed that immediately after exercise, expression of pro-inflammatory genes was significantly up-regulated in women in the luteal phase relative to the follicular phase and relative to men. Conversely, women in the luteal phase showed a strong trend towards down-regulation of anti-inflammatory genes relative to expression levels during the follicular phase and in men [3]. Similarly, Timmons and colleagues (2005) reported that exercise (90 min cycling at 65% max aerobic power) evoked higher numbers of circulating neutrophils, monocytes and lymphocytes during the luteal phase than during the follicular phase in women using oral contraceptives [4]. Together, these studies indicate that the immune system undergoes sex-specific responses to exhaustive exercise that are maximal during the luteal phase of the menstrual cycle, with the luteal phase biased towards pro-inflammatory and away from anti-inflammatory responses. This literature also highlights the need to consider hormone status in exercise immunological studies that include women. Indeed, many studies that did not control for menstrual cycle or the use of contraceptives at the time of testing have failed to find sex differences in immune responses to exercise, including measures of immune cell counts and functions [5–7], plasma cytokine levels [5, 8], and lymphocyte apoptosis [9].

While the literature suggests that the immune system undergoes sex-specific responses to exhaustive exercise, molecular studies at the transcriptional level are based on analysis of a relatively small number of genes [3], with to date no large scale microarray analysis of sex differences in the immune response. Thus, in the present study we used microarrays to assess the genome-wide transcriptional responses in men compared to women in the luteal phase of the menstrual cycle. Specifically, microarray analysis was combined with LPS stimulation of whole blood culture as a model for an in vivo infection [10, 11], allowing the transcriptional response to pathogen to be compared at multiple time points after exhaustive exercise (half marathon) in men and luteal-phase women. Since previous studies have demonstrated that some potentially important effects of exercise can only be detected in relation to pathogen stimulation of blood cells but not in native plasma nor in unstimulated blood cultures [12–18], we used whole blood cell culture with endotoxin stimulation to evaluate potential expression differences. In the present study, we have built on the initial findings and expanded analysis to specifically characterize sex-differences in the transcriptional immune responses of men and luteal-phase women to exhaustive exercise.

## Methods

### Subjects and sampling

Eight well-trained male athletes [34.8 ± 9.4 years, body mass index (BMI) 23.41 ± 2.2 kg/m2] and eight well-trained female athletes [38.5 ± 5.7 years, body mass index (BMI) 21.9 ± 1 kg/m2] participated in the study. Of these, four women who were in the second half of their menstrual phase and four randomly selected men were entered into microarray analysis. All athletes were non-smokers and none suffered from acute or chronic diseases or reported intake of medication or antioxidant supplements. All women in the study had regular menstrual cycles and none used oral contraception.

To identify menstrual cycle phase of the women, the hormonal status of women was determined by measuring plasma levels of estrogen, progesterone, leutenizing hormone (LH) and follicle stimulating hormone (FSH) using the ADVIA Centaur immunoassay system (Siemens Healthcare Diagnostics, Fernwald, Germany), following manufacturer’s instructions.

### Exercise program

The exercise program was an official half-marathon (21.1 km) under competition conditions. The run started at 10:00 AM on a cool and humid December day (1 °C) and took place on a hilly and demanding terrain. Prior to the half-marathon, all athletes performed an incremental exercise test on a treadmill (Saturn, HP Cosmos, Traunstein, Germany) to determine the running velocity at the individual anaerobic threshold (VIAT). VIAT was calculated by the method of [19].

### Whole blood culture and RNA isolation

K3-EDTA blood samples were taken before the half-marathon for baseline measures, and at several time points (30 min, 3 h, and 24 h) following completion of the half-marathon. Whole blood (2 × 9 ml) was cultured with vehicle or lipopolysaccharide (LPS) (10 ng/ml) for 1 h. LPS-stimulated and un-stimulated blood samples from both genders were transferred into PaxGene Blood RNA Tubes (PreAnalytiX/QIAGEN, Switzerland) and total RNA was isolated using the PaxGene Blood RNA kit (PreAnalytix/Switzerland) following the manufacturer’s protocol. Total RNA concentration was measured using spectrophotometry (Nanodrop 1000/Thermo Scientific) and RNA quality was assessed using a lab-on-a-Chip-System on the Bioanalyzer 2100 (Agilent/Germany) to ensure that samples used for microarray analysis had intact 18S and 28S ribosomal RNA peaks and minimal degradation.

### Gene expression profiling

Total RNA (100 ng) was linearly amplified and biotinylated using the GeneChip HT 3’IVT Express Kit (Affymetrix, UK) according to the manufacturer’s instructions. cRNA was hybridized onto Human Genome U219 Gene Chip-arrays (Affymetrix). Hybridization, washing, staining and scanning was performed automatically in a GeneTitan instrument (Affymetrix). Scanned images were subjected to visual inspection to control for hybridization artifacts and proper grid alignment, and were analyzed with AGCC 3.0 (Affymetrix) to generate CEL files.

### Bioinformatic data analysis

Microarray hybridizations were analyzed on the software platform R 2.12.0 with Bioconductor 2.10.0. Initially, the expression data from all chips were background corrected, quantile normalized and summarized with RMA (robust multichip average). A combined factor from treatment, time and gender was used to design a linear model which captures the influence on gene expression levels while using the individual donor as a random variable. A nonspecific filter based on overall variance was applied to remove non-informative genes before fitting the linear model. For the probesets that passed the filtering, the expression change relative to pre-exercise gene expression (e.g., delta: post-exercise expression minus pre-exercise expression) was calculated for at each time point (30 min, 3 h, 24 h) for each case. To identify sex-differences in gene responses, the delta at each time point was compared between men and women, in LPS-stimulated and LPS-unstimulated samples, with standard errors based on an empirical bayesian approach. *p* values were corrected for multiple testing using “Benjamini-Hochberg”, with statistical threshold for the corrected values set at *p* < 0.05.

Cluster analysis of genes showing significant sex specific patterns of response was performed in R 2.15.1. Signal intensities were scaled and centered and the distance between two expression profiles was calculated using euclidian distance measure. Hierachical cluster analysis was performed with average linkage. Heatmaps were generated with Bioconductor package geneplotter.

### Gene ontology and KEGG pathway analysis

In the lists of genes that were significantly differentially expressed with exercise and gender in our study, we conducted functional enrichment testing including Gene Ontology (GO) and Kyoto Encyclopedia of Genes and Genomes (KEGG; www.genome.jp/kegg/) pathways to determine the relative enrichment of genes with common or related functionalities to gain insight into biological processes mediated by both exercise and gender. This might give an overview which biological and molecular processes are responsible for the observed changes in transcription. A one-sided conditional hypergeometric test was used to analyze the lists of differentially regulated transcripts for over-representation of GO categories in the two GO main branches “biological process” and “molecular function”. GO categories with a *p* value of less than 0.01 were called significantly enriched. In the same way, the lists were analyzed for over-representation of known signal transduction and metabolic pathways from the KEGG database.

## Results

### Anthropometric and exercise data

The Anthropometric data has been presented in our previous publication [12] with all runners successfully completing the half-marathon race (21.1 km) with an average running time of 95.5 ± 8 min (86–116 min) for men and 114 ± 12 min (96–129 min) for women.

### Hormonal status

Basal hormone concentrations of plasma estrogen, progesterone, LH, and FSH were measured to confirm the phase of menstrual cycle of the women. The measurements confirmed that all female athletes were in luteal phase of their menstrual cycle (Table 1).

**Table 1:** Hormonal status of female athletes at baseline

LH (IU/l)	FSH (IU/l)	Estradiol (pmol/l)	Progesterone (pmol/l)
5.2 ± 4.07	5.1 ± 3.08	356.62 ± 339	4.48 ± 3,84

Values are in mean ± SD

### Blood cell count in response to exercise

Half-marathon running significantly increased total leukocyte counts for 3 h post exercise (*p* < 0.0001) in both male and female athletes, with no sex-specific differences in any immune cell population or in total leukocyte number. Neutrophil number and percentage were significantly increased at 30 min and 3 h post exercise. In parallel, the percentage of monocytes and lymphocytes was decreased at 30 min and 3 h post exercise, with lymphocyte percentage returning to pre-exercise levels at 24 h post exercise.

### Gene transcriptional and pathway regulation induced by exercise and differentially affected by gender (Time x Gender interaction)

Here, the changes in gene expression (up- or down regulation) from pre-exercise (t0) to post-exercise (t1,t2,t3) in male athletes in relation to the same status in female athletes are described (men_post-exercise – pre-exercise_) — (women_post-exercise – pre-exercise_). Following this algorithm, “upregulation” indicates a stronger transcriptional response (change from pre-exercise (t0)) in men relative to the response seen in women, and vice versa, “downregulation” indicates a weaker response in men relative to women. For example, a gene would be classified as “upregulated” if expression was (1) increased from pre-exercise levels in men with no change in women, or (2) decreased from pre-exercise levels in women with no change in men.

**Fig. 1:**
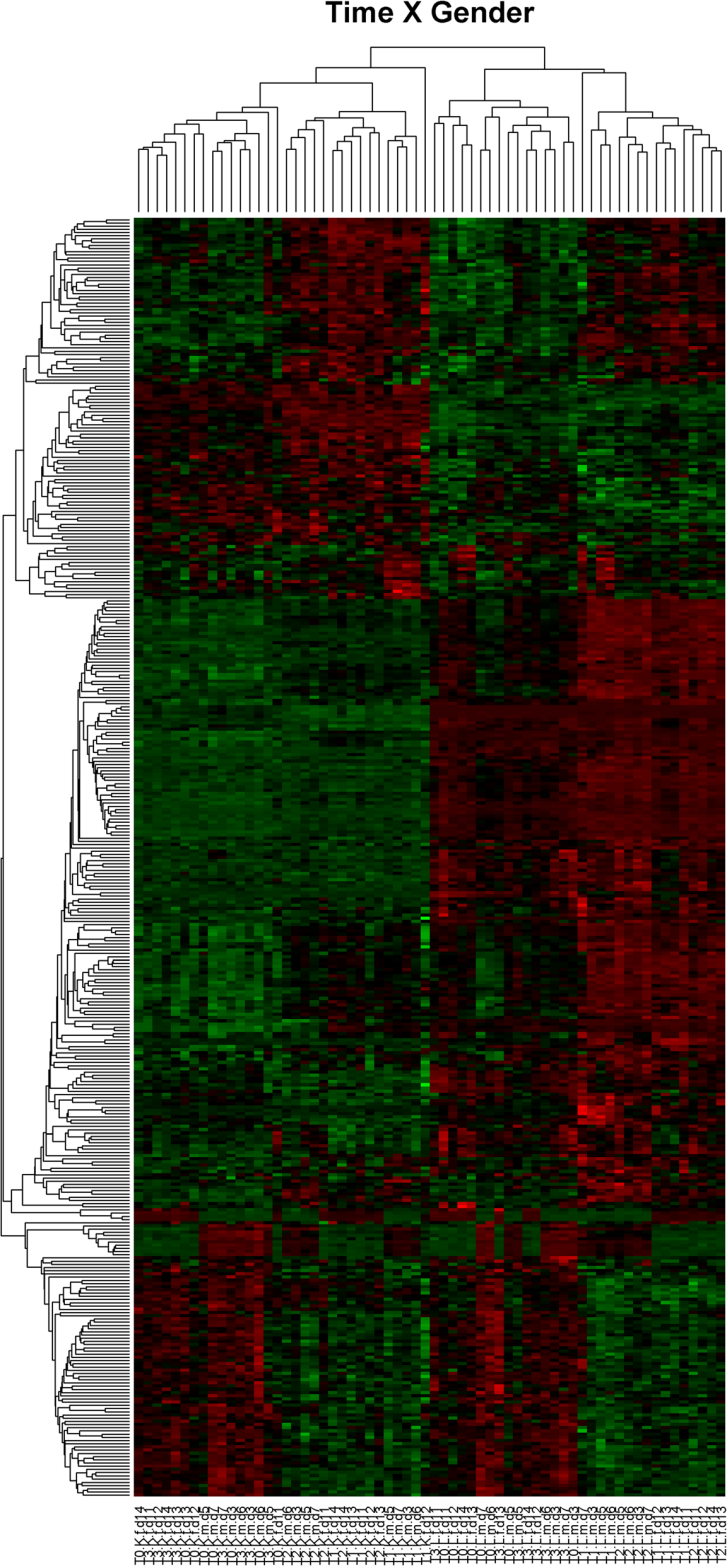
Hierarchical cluster analysis of all transcripts which showed an interaction effect between exercise and gender (t × g alg.). *Rows* correspond to probeset ids and each column (*x*-axis) represents a sample (a subject at the respective time point and treatment; T0, T1, T2, T3 = time points; *M* = Male, *F* = female, *K* = unstimulated culture, *L* = LPS stimulated culture, *d* = subject number). Expression profiles were scaled and centered, so that each transcript has an average expression of 0 and the same range between the lowest and highest values. High values are depicted in *red* while low values are shown in *green*

The global transcriptional profiles of LPS-stimulated and un-stimulated whole blood cultures from men and luteal-phase women in response to exhaustive exercise is shown in Fig. 1. In general, more genes were activated in men than luteal-phase women following exercise (Table 2). In addition, in both sexes, LPS-stimulated cultures showed a greater number of regulated transcripts compared to un-stimulated cultures at any time point (Table 2).

**Table 2:** The number of regulated transcripts for both sexes in each condition and time point

	T1	T2	T3
Men			
−LPS	3062	2544	5
+LPS	2695	3096	10
Women			
−LPS	2388	1736	11
+LPS	2279	1920	58

*T1:* 30 min post-exercise, *T2:* 3 h post-exercise, *T3:* 24 h post-exercise

*−LPS:* unstimulated cultures, *+LPS:* LPS-stimulated cultures

Several genes were significantly altered in terms of “time x gender (t × g) interaction”. Additional file 1: Table S1 shows a detailed list of regulated genes between men and women. According to this analysis, in LPS-stimulated cultures 191, 95 and 96 genes were differentially regulated between sexes at 30 min, 3 h and 24 h post exercise, respectively, while the number of differentially regulated genes for unstimulated cultures was lower; 68, 62 and 16 genes at 30 min, 3 h, and 24 h post exercise, respectively. Of these, genes which were relevant for immune system/inflammatory response, metabolic response, DNA methylation, thrombotic system and brain function became pronounced. Table 3 summarizes the most differentially and significantly regulated genes between sexes at each time-point (t × g alg.) for each of the culture conditions (e.g., LPS-stimulated and unstimulated). These included 10 most significantly regulated genes from each condition × each time point (e.g., T1-T0 men-control in relation to T1-T0 women-control).

**Table 3:** Most differentially regulated genes between sexes in each time-point (t × g alg.) in both cultures.The fold change calculated based on log 2 (men_post ‐ exercise – pre ‐ exercise_) — (women_post ‐ exercise – pre ‐ exercise_)

Gene ID	Gene symbol	Log2 FC inUnstimulated culture			Log2 FC in LPS-stimulated culture		
		T1-T0	T2-T0	T3-T0	T1-T0	T2-T0	T3-T0
11721945_a_at	MRVI1	1.967	0.428	0.424	1.917	0.866	1.298
11716974_a_at	PDK4	1.817	0.527	1.132	0.328	0.109	0.075
11757733_s_at	XIST	1.590	1.0798	−0.715	0.1970	0.277	−0.549
11721216_s_at	TMEM106B	1.477	−0.309	0.474	1.912	0.735	1.024
11764064_s_at	CYorf15B	−1.964	−1.345	−0.306	−1.848	−1.350	0.025
11754026_a_at	IL8	−1.795	−2.040	−0.863	−0.527	0.453	0.133
11736405_a_at	DNMT1	−1.481	−1.246	−0.369	0.478	−0.292	0.395
11726814_x_at	KDM5D	−1.405	−0.810	−0.131	−0.922	−0.988	−0.026
11729424_s_at	CCRL2	0.841	1.282	0.764	0.897	0.736	0.557
11757485_x_at	FAM129B	0.533	1.269	0.744	0.532	0.459	0.387
11759616_at	VASH1	0.567	1.053	0.843	0.428	0.072	0.191
11720029_a_at	LDLR	0.123	1.027	0.910	0.136	0.000	0.626
11744660_s_at	CCL4L1///CCL4L2	−1.334	−1.948	−0.062	−0.047	0.183	−0.015
11723679_s_at	CD69	−0.337	−1.436	−0.780	−0.346	−0.102	−0.144
11763717_a_at	CDKN1C	−0.754	0.253	1.168	−1.037	−0.917	0.202
11755211_a_at	DICER1	−0.043	0.058	1.042	−0.152	−0.165	0.214
11738041_x_at	TNIP3	−0.025	0.020	−0.028	3.371	1.228	0.582
11722015_at	OLR1	−0.081	−0.165	−0.061	2.265	2.042	1.591
11756590_a_at	KMO	−0.338	−0.010	−0.287	1.979	1.527	1.174
11754526_a_at	PTGES	0.154	0.116	0.019	1.954	1.192	1.158
11728477_at	CXCL3	−0.136	−0.008	−0.095	−2.446	−0.046	−1.031
11758472_s_at	RICTOR	−1.204	−0.159	−0.631	−2.035	−0.308	−0.766
11732719_at	EREG	0.049	0.433	−0.041	−1.819	−1.211	−0.983
11735242_s_at	ZC3H12C	0.020	0.344	−0.114	1.948	1.643	0.715
11727181_a_at	SKIL	0.226	0.010	0.3023	0.869	1.526	0.598
11720298_at	CXCL10	0.016	−0.056	0.109	1.500	1.432	1.340
11746463_a_at	IL6	0.313	0.259	0.373	1.063	1.228	1.165
11736090_a_at	OLIG2	−0.384	−0.563	−0.365	−1.087	−1.499	0.054
11757044_x_at	C7orf68	−0.539	−0.187	−0.154	−1.008	−1.426	−0.203
11716496_s_at	CHMP4B	0.6431	−0.138	0.267	1.844	1.212	1.720
11718394_at	JUN	0.431	−0.152	0.560	1.154	0.184	1.562
11740393_at	TNFRSF9	0.063	0.452	0.648	0.967	1.144	1.536
11752419_a_at	HIVEP1	0.170	−0.106	−0.079	1.826	1.184	1.416
11718759_x_at	HIF1A	0.370	−0.333	−0.160	0.263	1.077	1.395
11721034_at	PPP1R3B	0.584	0.183	−0.198	−1.145	−0.330	−1.121
11721542_a_at	MARCH9	−0.309	0.254	−0.039	−0.614	−0.808	−1.118
11718452_at	AXIN2	−0.449	−0.155	−0.886	−0.555	−0.551	−1.094
11720062_s_at	IER3	1.038	0.375	0.825	0.092	0.048	0.135
11750167_a_at	CAPN2	−0.999	−0.637	−0.153	−0.630	−0.144	−0.318
11717656_a_at	SPRED2	1.066	0.514	0.309	0.979	0.953	0.038
11719154_a_at	DRAM1	0.395	−0.095	−0.152	1.344	1.036	0.927

Among the inflammatory genes which were differentially regulated between sexes in response to exercise, TNIP-3 (*p* = 2.39E–17), IL-6 (*p* = 9.03E–43), IL-8 (*p* = 5.93E–25), CXCL10 (*p* = 1.84E–17), CXCL3 (*p* = 2.37E–15), CCR3 (*p* = 5.02E–08), CAPN2 (*p* = 1.77E–05), and CD69 (*p* = 7.76E–11) were most prominent. TNIP3, a gene with potential anti-inflammatory function, was strongly induced by exercise in LPS-stimulated cultures of men, while women showed only a trend toward up-regulation. Men showed higher expression of LPS-stimulated IL-6 mRNA than women. While IL-8 did not change in men, it was significantly up-regulated in unstimulated cultures of women. LPS-stimulated (but not unstimulated) expression of CXCL10 mRNA was significantly and more strongly down-regulated in women compared to men (sixfold vs fourfold, respectively).

In addition, thrombotic system-related genes, MRVI1 and PLAU, were significantly induced by exercise with more pronounced induction in men.

Furthermore, genes relevant for brain function were also differentially regulated between sexes. Oligodendrocyte transcription factor 2 (OLIG2) was significantly down-regulated by exercise but only in LPS-stimulated cultures and only in men (*p* = 6.85E-09). TMEM106B (*p* = 0.00043248) was significantly (LPS) or mildly (no LPS) (P = 0.00043248) up-regulated in men while it was significantly (no LPS) or mildly (LPS) down-regulated in women. KMO (*p* = 9.25E–14) was significantly downregulated in LPS-stimulated cultures of women, but not men. DDIT4 (*p* = 8.08E–24) showed markedly downregulation 3 h post exercise in unstimulated cultures in men significantly more than in women.

Growth factors VEGFA, IGF1R, and IGF2R were also sex-specifically induced by exhaustive exercise.

A sex-specific response of some of the above mentioned genes such as IL-6 and IL-8 has been previously validated by qRT-PCR [1].

KEGG pathway analysis was used to assign the differentially regulated genes between sexes into functional categories in both LPS-stimulated and un-stimulated cultures. A detailed list of pathways which were significantly over-represented in each group is shown in Table 4. KEGG pathways were activated for sex differences in LPS-stimulated only but not in un-stimulated cultures. The toll-like receptor signaling pathway (*p* = 0.000) and cytosolic DNA-sensing pathway (*p* = 0.004) were the only over-represented pathways for differentially regulated genes between sexes at 30 min post exercise. Four KEGG pathways were differentially over-represented for sex differences at 3 h post exercise and “Epithelial cell signaling in Helicobacter pylori infection” (*p* = 0.010) was the only significantly regulated pathway between sexes 24 h after exhaustive exercise (Table 4).

**Table 4:** The KEGG pathways significantly over-represented between male and female athletes in LPS-stimulated cultures following exhaustive exercise

KEGG pathways		Count	*p* value
30 min post Ex.	Toll-like receptor signaling pathway	7	0.000
	Cytosolic DNA-sensing pathway	4	0.004
3 h post Ex.	Cytosolic DNA-sensing pathway	4	0.000
	Toll-like receptor signaling pathway	5	0.001
	RIG-I-like receptor signaling pathway	4	0.001
	Amoebiasis	4	0.004
24 h post Ex.	Epithelial cell signaling in Helicobacter pylori infection	3	0.010

## Discussion

In this study, we present a detailed analysis of sex-specific changes in transcriptional responses following exhaustive exercise in LPS-stimulated and un-stimulated whole blood cultures, as a follow up to our previous study [12]. Here, we found that men expressed higher numbers of activated genes at each time point after exercise even though luteal-phase women showed a greater extent of pathway activation than men. Our current study together with the finding of our previous study [3] demonstrates that women in the luteal phase of their menstrual cycle show a different regulation following exercise as compared to men or women in their follicular phase. It should be noted that the sex differences in gene induction cannot be attributed to exercise-associated cellular shifts, since men and women exhibited virtually identical shifts in circulating cell numbers, probably reflecting the fact that the relative intensity of exercise was identical for men and women in this competitive situation.

We demonstrated that exercise induced sex-specific expression of genes encoding products involved in innate immune/inflammatory responses, metabolic responses, the cell cycle, brain function, apoptosis, and regulation of transcription. Below, we discuss the sex-specific regulation of inflammatory response genes and genes relevant to brain function.

### Differential activation of signaling pathways between sexes

A prime finding of our analysis for sex differences is the observation that five KEGG pathways showed sex-specific responses to endotoxin after exhaustive exercise, including inflammation-related pathways TLRs, cytosolic DNA sensing, and RIG-I like receptors (Table 4). These pathways all serve to organize the primary reaction of the innate immune system to microbes of bacterial or viral origin. Of these activated pathways, our data revealed that the TLR signaling pathway was the most prominent with seven genes showing sex-specific regulation already at 30 min post exercise. Cytosolic DNA sensing receptor and RIG-I like receptor signaling pathways were the next two differentially overrepresented pathways between sexes (Table 4).

TLRs are external (e.g., TLR3,4) or internal (e.g., TLRs 3,7,8) receptors for bacterial cell wall components like LPS or microbial nucleic acids or analogues and induce synthesis of inflammation related cytokines (IL-1, IL-6, TNF-α) and/or type I interferons (IFN-β1 and IFN-α) [20]. DNA sensing and RIG-I like receptor pathways are triggered mainly by virus derived nucleic acids and lead to the production of type I interferons, but also other inflammatory cytokines including activation of the necessary machinery for protein synthesis. Excessive activation of TLRs is implicated in the pathogenesis of infectious and inflammatory diseases [21–23]. A recent work by Khan et al (2010) showed higher response by TLR-7 and TLR-8 but not TLR-4 and TLR-3 in healthy females as compared with males [20].

The differential pathway activation between sexes as shown by the above mentioned KEGG analysis, confirms that there are significant differences between the reaction of men vs women in luteal phase in the setting of exercise plus pathogen stimulation.

KEGG pathway analysis revealed differences only in LPS-stimulated cultures, underlining the benefit of testing the interaction of exercise with pathogen contact. Appearance of sex-specific differences in KEGG pathways of pathogen receptors TLRs, DNA sensing and RIG-I like receptors, highlights this message and is shown here for the first time.

Our data suggest that exercise interacts with early steps of the pathogen response, involving at least three major pathways of innate immunity, as discussed in the next section.

### Exercise and the inflammatory response genes

In the present study, we found a bias towards less anti-inflammatory regulation in luteal-phase women as compared to men, confirming our previous finding [3]. It is intriguing that this bias was a combined action of lower expression of anti-inflammatory genes and higher expression of pro-inflammatory genes in women.

TNIP-1 and TNIP-3 (TNFAIP3 interacting protein-1, -3) are potent multiple action inhibitors within the TLR pathway [24]. Their activation became only apparent in LPS-stimulated cultures, and men showed highly significant activation while women showed only a trend. Similarly, the central protective gene IL-6, the tumorigenesis controlling gene HIVEP1[25], and SPRED2 [26] which are all clearly anti-inflammatory in their effects were significantly more highly expressed in men than women.

On the other hand, several genes with prominent pro-inflammatory function such as IL-8, CXCL3, CCR3, CAPN2, and CD69 were also differentially regulated between sexes in response to exercise. While IL-8, a major player in innate immunity, did not change in men, it was significantly up-regulated in unstimulated cultures of women. Similarly, CXCL3, a strongly inflammatory chemokine, was significantly down-regulated in men while significantly up-regulated in women. Contrasting this, LPS-stimulated (but not unstimulated) expression of CXCL10 mRNA was significantly and more strongly down-regulated in women compared to men (sixfold vs fourfold, respectively). LPS-stimulated and unstimulated expression of CCL5 (RANTES) mRNA was also significantly down-regulated following exercise but with no differences between sexes. It should be noted that all chemokines mentioned above have potent pro-inflammatory activities in addition to their chemotactic properties and have been implicated in the progression of several inflammatory and autoimmune diseases [27].

CD69, a lymphocyte activation molecule, which is also involved in the pathogenesis of chronic inflammation [28], was also significantly down-regulated in both sexes with men having more pronounced regulation than women. A significant down-regulation of CD69 mRNA after 60 min of post-exercise recovery had also been detected in response to an acute bout of 30 min exercise [29].

Taken together, in exercise induced gene regulation, we see a sex/menstrual phase specific bias towards less anti-inflammatory regulation in luteal-phase women as compared to men. The question regarding what is behind these sex/menstrual phase-specific differences in gene expression is not easy to answer. It seems that following exhaustive exercise there is a substantial change in gene expression in the direction of an increased pro-inflammatory state in the luteal phase of female athletes. Previously, Lynch et al have showed that men and women differentially regulate the IL-1/IL-1ra system in PBMCs, with women showing more pro-inflammatory regulation in the luteal phase [30]. To answer this question, further studies including replication and much studies in animal models are needed.

Using qRT-PCR, we have previously shown the identical responses from some of the above mentioned genes (e.g., IL-6, IL-8) in the blood culture of same exercising individuals [1], confirming first, the results of the present microarray study and second, an inflammatory bias of women in their luteal phase.

### Exercise differentially modifies the expression of genes relevant for brain function and structure

**Fig. 2:**
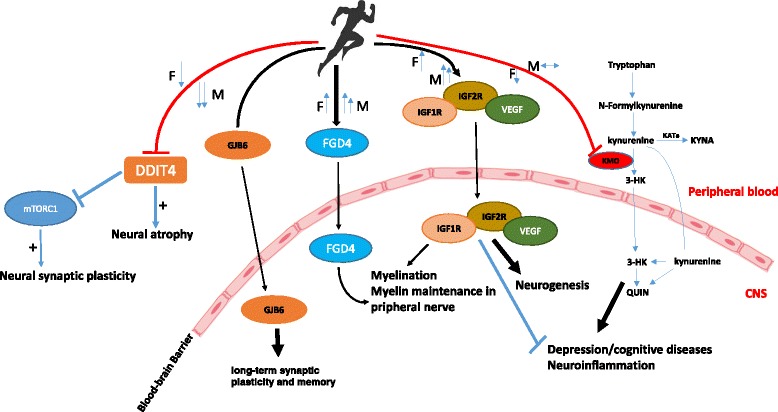
Possible mechanisms for neuroprotective function of exercise and differences between sexes. DDIT4, a gene which is associated with promotion of neuroal atrophy and blocks mTOR signaling pathway to neural plasticity, was down-regulated through exercise, more so in men than in women (in luteal phase). Conversely, KMO, a pivotal enzyme of the kynurenine pathway of tryptophan metabolism, associated with induction of depression, was down-regulated in women only. Growth factors VEGFA, IGF1R, and IGF2R were also induced by exercise with minor differences between sexes. Finally, FGD4 mRNA, a myelination inducing gene, was significantly enhanced by exercise in both sexes with men showing more pronounced regulation. Overall, the observed gene expression regulations may be interpretable as neuroprotective effects of exercise

Several genes which are relevant for brain function were also differentially and significantly regulated between sexes in response to exhaustive exercise. In the present study we found a sex-specific regulation of KMO (kynurenine 3-monooxygenase) in response to exhaustive exercise (Fig. 2). KMO was significantly downregulated in LPS-stimulated cultures of women, but not men. KMO is a pivotal enzyme of the kynurenine pathway of tryptophan metabolism and has been associated with schizophrenia and induction of depression [31, 32]. In the presence of KMO, kynurenine is converted to 3-HK, resulting in the infiltration of 3-HK to brain which can subsequently cause neuroinflammation. In the absence of KMO, kynurenine is converted to kynurenic acid (KYNA) which has potent anti-inflammatory and protective properties [33]. Thus, sex-specific inhibition of LPS-stimulated KMO mRNA by exercise could be a modulatory signal for women who are already in an inflammation prone phase (luteal phase). This is the first report of KMO regulation through exercise. Just recently, it was shown that exercise-induced increases in kynurenic acid via KAT enzymes in muscle are suggested to be associated with brain protection [34].

DDIT4 (DNA damage inducible transcript 4), an inhibitor of mTOR signaling, is another interesting molecule involved in synaptic loss, neural atrophy and depressive behavior [35]. Following slight non-significant induction at 30 min post exercise, it was markedly downregulated 3 h post exercise in unstimulated cultures in men significantly more than in women. Since mTOR plays a pivotal role in synaptic plasticity, inhibition of DDIT4 by exercise may also be considered as a possible mechanism for the beneficial and antidepressive functions of exercise.

Four further genes (VEGFA, IGF1R, IGF2R, and FGD4) involved in peripheral and/or central nerves growth, myelination as well as learning [36, 37], were also strongly induced by exercise in LPS-stimulated and unstimulated cultures. Three of these (IGF1R, IGF2R, and FGD4) showed significant differences between sexes with women having slightly less induction than men (Fig. 2).

Although the beneficial effects of exercise- regulated expression of leukocyte derived factors on brain function has not been demonstrated extensively, it has previously been suggested that the peripheral increase in the growth factors such as BDNF, IGF-1, and VEGF seems to be essential for exercise-induced neurogenesis and improved memory [36]. Recent attempts to identify cellular players have highlighted the role of soluble factors in the blood circulation and immune cells in regulating hippocampal neurogenesis/neuroplasticity in adulthood [38]. Adult hippocampal neurogenesis is markedly decreased in mice lacking a fully functioning immune system and could not be enhanced by environmental enrichment [39]. A very recent report showed that voluntary exercise-induced blood-borne Ly6C(hi) monocytes rescues neurogenesis and the behavioral deficits in antibiotics-treated mice [40].

It should be noted that the findings of our present study are correlative and may not underlay the full neuroprotective benefits observed following exercise intervention. However, exercise-induced up-or down-regulation of the above mentioned genes may bring some insights into the mechanisms by which how exercise-induced leukocyte/leukocyte genes could exert beneficial function on brain. Here, we propose that exercise-induced leukocyte-derived growth factors as well as factors such as FGD4 may cross the blood–brain barrier (BBB) and drive beneficial effect on brain function (Fig. 2).

## Conclusions

Taken together, the most prominent finding of this analysis is that there were sex-specific differences in activation of inflammation-related pathways TLRs, cytosolic DNA sensing, and RIG-I like receptors. Individual considerations of inflammation related genes like TNIP-1, TNIP-3, IL-6, HIVEP1, CXCL3, CCR3, IL-8, and CD69 confirms that there was a bias toward less anti-inflammatory activation in the women tested who were all in the luteal phase of their menstrual cycle. Furthermore, men showed higher activation of antithrombotic genes MRVI1 and PLAU through exercise while women had a higher baseline. Finally, exercise also modified a set of genes related to brain function and structure (KMO, DDIT4, VEGFA, IGF1R, IGF2R, and FGD4), with significant differences between sexes. Sex-specific regulation of leukocyte genes in response to acute exhaustive exercise might be contributed in exercise-derived benefits to brain function (Fig. 2).

## Additional file

Additional file 1: Table S1. The list of differentially regulated genes between sexes in response to exercise (t × t interaction) in both stimulated and un-stimulated whole blood cultures. (XLSX 76 kb)

## Abbreviations

CCR3: C-C motif chemokine receptor 3
CXCL3: C-X-C motif chemokine ligand 3
DDIT4: DNA-damage-inducible transcript 4
HIVEP1: Human immunodeficiency virus type I enhancer binding protein 1
KMO: Kynurenine 3-monooxygenase
RIG-I-like receptors: Retinoic acid-inducible gene I-like receptors
TLRs: Toll-like receptors
TNIP3: TNFAIP3 interacting protein 3
